# Performance characteristics of polymer photovoltaic solar cells with an additive-incorporated active layer

**DOI:** 10.1186/1556-276X-7-56

**Published:** 2012-01-05

**Authors:** Hyomin Kim, Sunseong Ok, Hyunhee Chae, Youngson Choe

**Affiliations:** 1 Department of Chemical Engineering, Pusan National University, Busan, 609-735, South Korea

**Keywords:** bulk heterojunction, power conversion efficiency, polymer solar cell, excitons

## Abstract

We have investigated the performance characteristics of bulk-heterojunction polymer solar cells based on poly(3-hexylthiophene-2,5-diyl) and [6,6]-phenyl C_61 _butyric acid methyl ester by adding 1,8-octanedithiol as a processing agent in an active layer. The effects of the additive, 1,8-octanedithiol, on the device performance parameter characteristics have been discussed. The current density-voltage measurements, UV-Vis absorption spectra, X-ray diffraction spectra, and scanning probe microscope images have been used to discuss the performance characteristics of polymer solar cells.

## Background

Clean and renewable energies have been considerable issues in the last decade. For this reason, organic photovoltaic cells have been attractive devices as next-generation substitute energy sources [1-4]. Currently, the power conversion efficiencies of organic photovoltaic cells have been steadily improved around 6% through polymer solar cells [5]. There have been reports that polymer solar cells have many advantages of cost-effectiveness in the fabrication process, and the mechanical flexibility and polymeric materials provide a wide field of applications [6,7].

Bulk-heterojunction [BHJ] solar cells, based on phase-separated blends of polymer semiconductors and fullerene derivatives, typically consist of a conjugated polymer, poly(3-hexylthiophene-2,5-diyl) [P3HT] as an electron donor, and fullerene derivatives, [6,6]-phenyl C_61 _butyric acid methyl ester [PCBM] as an electron acceptor [8-12]. Especially, P3HT has attracted lots of interest due to its high crystallinity and self-assembling property. In supporting P3HT crystallite formation, PCBM should be dispersed between P3HT chains [13]. For this, thermal and solvent annealing can be used to improve their roles between P3HT and PCBM [14,15]. Recently, a small volume ratio of additives such as 1,8-octanedithiol has been incorporated into the P3HT:PCBM system to improve the interactions between P3HT and PCBM [16].

In this work, we have fabricated BHJ solar cells based on P3HT and PCBM, which were dispersed using a single solvent, chlorobenzene and 1,2-dichlorobenzene. The effects of the additive, 1,8-octanedithiol, on the performance characteristics of polymer solar cells have been investigated. The results of current density-voltage [*J*-*V*] measurements, UV-Visible [UV-Vis] absorption spectra, X-ray diffraction [XRD] spectra, and scanning probe microscope [SPM] images will be intensively used to discuss the performance characteristics of polymer solar cells fabricated in this study.

## Methods

BHJ films were prepared via a solution process. P3HT (Rieke Metals, Inc., Lincoln, NE, USA) and PCBM (Nano-C, Westwood, MA, USA) with a 1:1 wt/wt ratio was dissolved in chlorobenzene and 1,2-dichlorobenzene to make a 2.4 wt.% solution. The blend solution was stirred for 24 h at 40°C in a shaking incubator. 1,8-Octanedithiol (formula C_8_H_18_S_2_, molecular weight 178.36 g/mol, boiling point 269°C to 270°C, density, 0.97 g/mL at 25°C, Sigma-Aldrich Corporation, St. Louis, MO, USA) and 1,8-diiodooctane (formula C_8_H_16_I_2_, molecular weight 366.02 g/mol, boiling point 167°C to 169°C, density 1.84 g/mL at 25°C, Sigma-Aldrich Corporation) were selected as additives, and 2.5 vol.% additives were then added into the base solution. The solution containing a mixture of P3HT:PCBM with processing additives was stirred for 10 min. Polymer solar cells were fabricated on the pre-patterned indium tin oxide [ITO] glass substrate. Poly(3,4-ethylenedioxyhiophene):poly(styrenesulfonate) [PEDOT:PSS] was spin-coated onto the ITO substrate at 3,000 rpm for 30 s, and the prepared thin film was then baked at 120°C for 10 min on a hot plate in air. The prepared solution was spin-coated onto the PEDOT:PSS layer at 1,000 rpm for 30 s, and then, the spin-coated thin film was dried in a Petri dish. As a final step, an Al electrode was deposited onto the spin-coated layer by thermal evaporation. The fabricated devices were annealed at 120°C for 30 min. An active area of the device, 2 mm × 2 mm in dimension, was made using a shadow mask. The *J*-*V *and power conversion efficiency (*η*_e_) characteristics were measured using a 2400 multi-source meter unit (Keithley Instruments, Inc., Seoul, South Korea). A xenon lamp (100 mW/cm^2^) was used as a light source, and the light intensity has been measured by a silicon photodiode calibrated for an AM 1.5 spectrum. The absorption spectrum were taken using an Optizen 2120UV spectrophotometer (Mecasys Co., Ltd., Daejeon, South Korea); XRD images were obtained using a high-resolution X-ray diffractometer (Philips, Amsterdam, The Netherlands); and SPM images were obtained using a SPM (Multimode, Digital Instruments, Inc., Tonawanda, NY, USA).

## Results and discussion

**Figure 1:**
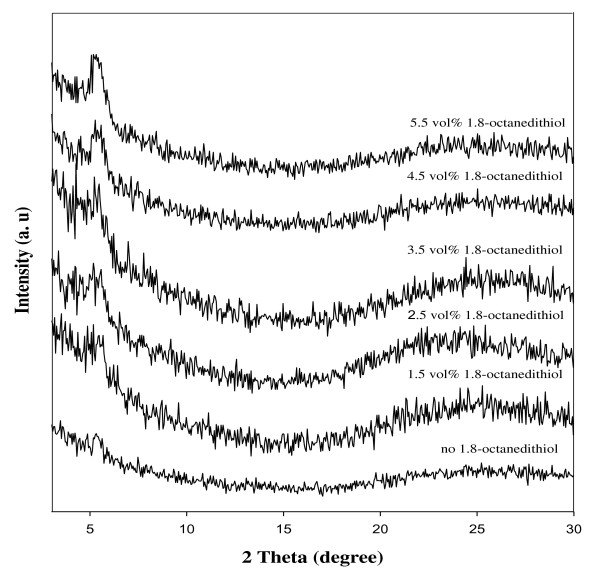
**XRD spectra of the devices solution-processed with chlorobenzene and different amounts of 1,8-octanedithiol**.

The XRD spectrum of active layers, P3HT:PCBM films, are shown in Figure 1. When the processing additive, 1,8-octanedithiol, was used, peak intensities were much higher than those of the films without 1,8-octanedithiol, and this implies that the P3HT:PCBM films possess a crystalline nature and that highly ordered structures are formed in the films using a processing additive. The crystallinity of P3HT in the films significantly increases with the presence of 1,8-octanedithiol. It implies that the interaction between P3HT is stronger, and the size distribution of P3HT crystals is broader with an increasing amount of 1,8-octanedithiol. The processing additive, 1,8-octanedithiol, could provide a stronger driving force for polymer aggregation. A highly ordered structure in P3HT:PCBM films can provide short pathways to benefit the carrier mobility.

**Figure 2:**
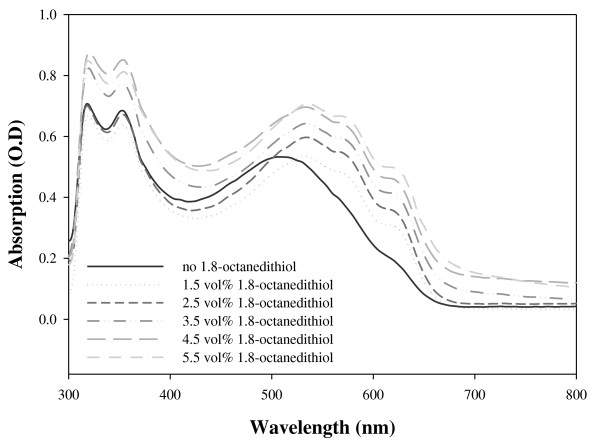
**UV-Vis absorption spectra of the devices solution-processed with chlorobenzene and different amounts of 1,8-octanedithiol**.

Absorption spectra of active layers are shown in Figure 2. As the amount of 1,8-octanedithiol was increased in the BHJ film formation process, the absorption intensities were increased. P3HT:PCBM composite films processed with 1,8-octanedithiol have shown three dominant features in absorption: two peaks at 510 and 550 nm and one shoulder at 610 nm appeared due to strong interchain interactions. When adding 1,8-octanedithiol, the absorption band of P3HT:PCBM composite film peaks are red-shifted, and the intensity of the absorption band only increased with the increasing amount of 1,8-octanedithiol. Such a shift on the absorption peak is associated with *π*-π* transition, indicating that the P3HT chains interact more strongly. At the presence of PCBM, a uniform dispersion of polymer aggregates can be obtained. Therefore, it is considered that the addition of 1,8-octanedithiol helps the crystallization of P3HT as observed by the absorption spectrum.

**Figure 3:**
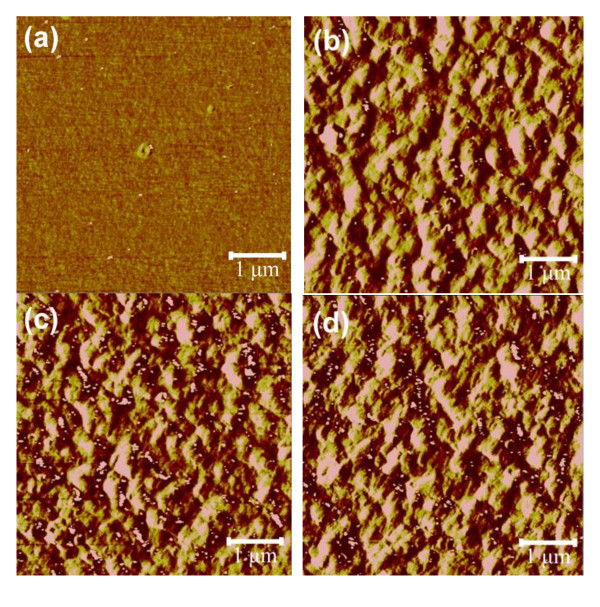
**SPM images of P3HT:PCBM films formed using chlorobenzene with different amounts of 1.8-octanedithiol. Chlorobenzene alone**. (**a**), chlorobenzene with 1.5 vol.% of 1.8-octanedithiol (**b**), chlorobenzene with 3.5 vol.% of 1.8-octanedithiol (**c**), and 1,2-dichlorobenzene with 5.5 vol.% of 1.8-octanedithiol (**d**).

From the SPM images, as shown in Figure 3, the growth of polymer aggregates or clusters is clearly seen. The aggregate size gets bigger with the increasing amount of 1,8-octanedithiol, consistent with the higher crystallinity observed in the XRD spectrum when increasing the amount of 1,8-octanedithiol. The roughness value and aggregate size are very important because of the fact that the exciton diffusion length in a polymer system is about 5 to 10 nm. Therefore, it is necessary to maintain a proper size of the polymer aggregate because of an efficient dissociation of excitons generated in the films to achieve higher efficiency. A finely dispersed structure is observed when there is no 1,8-octanedithiol. Thin fibrillar structures appear when the amount of 1,8-octanedithiol reaches 1.5 vol.%, as shown in Figure 3, and Figure the P3HT domain grows bigger when more than 1.5 vol.% 1,8-octanedithiol is added.

**Figure 4:**
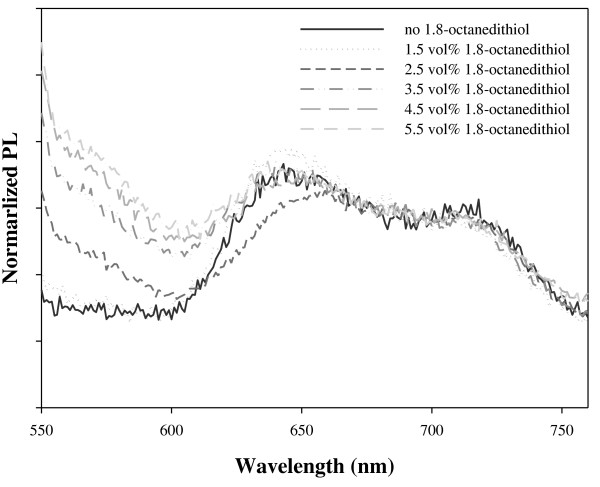
**PL spectra of the devices solution-processed with chlorobenzene and different amounts of 1,8-octanedithiol**.

The photoluminescence [PL] spectra of active layers are shown in Figure 4. The PL intensity increased in the wavelength range of 550 to 650 nm with the increasing amount of 1,8-octanedithiol. A high PL intensity indicates that not all excitons generated on one polymer within the film reached the interface of the other polymers [17]. When the conjugation length increases or when the domain size of P3HT increases, the PL intensity of P3HT increases [18]. An increase in the PL intensity suggests that PCBM is not close enough to contact with P3HT to undergo a charge transfer, and the interface area between P3HT and PCBM is decreasing [19]. It is observed that more severe phase separation occurred when more 1,8-octanedithiol is added. It appears that after the exciton dissociates at the P3HT:PCBM interface, an efficient carrier collection is required for a high performance of the device. When adding an additive, carrier transport pathways, associated with the crystallinity of P3HT, can be formed well. Through the analysis results of the UV-Vis absorption and PL spectrum, it can be considered that there is a proper point to dissociate the exciton to achieve higher device performance. In addition, the growth of P3HT domains is consistent with the PL spectra results showing that the interface area of P3HT and PCBM is decreasing and also consistent with the XRD spectra (Figure 1), showing wider polymer domain distributions.

**Figure 5:**
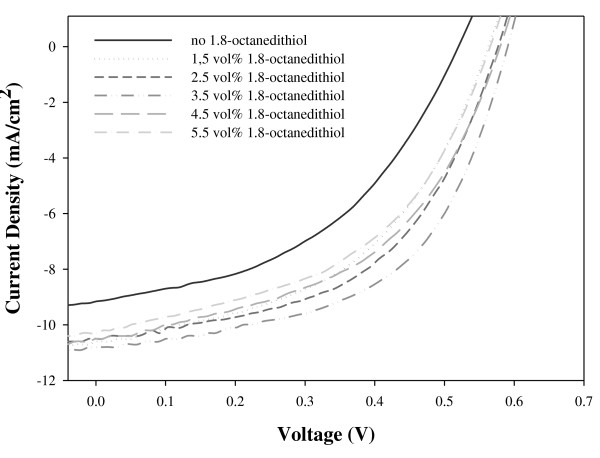
***J*-*V *curves of the devices solution-processed with chlorobenzene and different amounts of 1,8-octanedithiol**.

**Figure 6:**
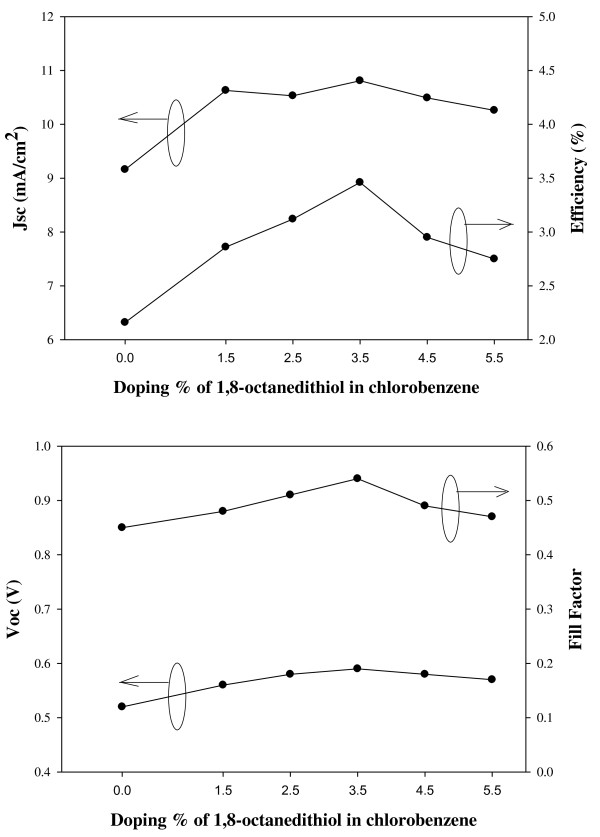
**Photovoltaic response**. Photovoltaic response of solar cell devices with chlorobenzene and different amounts of 1,8-octanedithiol, *J*_sc_, PCE, *V*_oc_, and FF.

The *J*-*V *curves of devices, which are solution-processed using different amounts of 1,8-octanedithiol, are shown in Figure 5. By introducing a small amount of the additive to a solution-processed active layer, the *J*-*V *characteristics of the active layer were improved, and consequently, higher power conversion efficiency [PCE] of the device was obtained as shown in Figure 6. The values of a short-circuit current density [*J*_sc_], a fill factor [FF], an open-circuit voltage [*V*_oc_], and PCE were all improved as 1,8-octanedithoil was added until 3.5 vol.%. However, when adding over 4.5 vol.% of 1,8-octanedithiol, the values of all characteristic parameters were decreased. As a result, when chlorobenzene as a solvent and 3.5 vol.% 1,8-octanedithiol as an additive were employed in a solution process, the performance characteristics of the device were significantly improved, showing that *J*_sc _= 10.81 mA/cm^2^, FF = 0.54, *V*_oc _= 0.59 V, and PCE = 3.46%. Even though the absorption intensity and crystallinity are increased, the PL intensity also increased. Because of this reason, the film with 3.5 vol.% of 1,8-octanedithiol exhibited the best device performances in this work.

## Conclusions

The performance characteristics of BHJ polymer solar cells based on P3HT and PCBM can be improved by introducing a processing additive, 1,8-octanedithiol, to a solution-based film formation process, and an optimized amount of 1,8-octanedithiol can be determined. As the amount of 1,8-octanedithiol was increased, the intensity of the UV-Vis absorption and the crystallinity of P3HT significantly increased, and the PL intensity also increased simultaneously, consequently exhibiting the improved performances of the BHJ polymer solar cells. By employing the processing additive, 1,8-octanedithiol, the PCE was increased from 2.16% to 3.46% in this study.

## Abbreviations

BHJ: bulk heterojunction; ITO: indium tin oxide; PCBM: [6,6]-phenyl C_61 _butyric acid methyl ester; PCE: power conversion efficiency; PEDOT:PSS: poly(3,4-ethylenedioxythiophene:poly(4-styrenesulfonate); PL: photoluminescence; P3HT: poly(3-hexylthiophene-2,5-diyl); SPM: scanning probe microscope; XRD: X-ray diffraction.

## Competing interests

The authors declare that they have no competing interests.

## Authors' contributions

HK and HC planned the experiment, taking part in drawing the outlines of the manuscript. SO performed the experimental analyses. YC conceived the study and joined the experimental design and coordination. All authors read and approved the final manuscript.

## Authors' information

HK, SO, and HC are students of a Master's course and YC is a professor in the Chemical Engineering Department of Pusan National University, South Korea.
